# Two novel species of *Colletotrichum* (*Glomerellaceae*, *Glomerellales*) from Sichuan Province, China

**DOI:** 10.3897/mycokeys.141.209472

**Published:** 2026-09-30

**Authors:** Feihu Wang, Feng Liu, Xiulan Xu, Chunlin Yang, Yinggao Liu

**Affiliations:** 1 College of Forestry, Sichuan Agricultural University, Chengdu, Sichuan, 611130, China College of Forestry, Sichuan Agricultural University Chengdu China; 2 Forest Ecology and Conservation in the Upper Reaches of the Yangtze River Key Laboratory of Sichuan Province, Chengdu 611130, Sichuan, China Forest Ecology and Conservation in the Upper Reaches of the Yangtze River Key Laboratory of Sichuan Province Chengdu China; 3 Sichuan Mt. Emei Forest Ecosystem National Observation and Research Station, Chengdu 611130, Sichuan, China Sichuan Mt. Emei Forest Ecosystem National Observation and Research Station Chengdu China; 4 Forestry Research Institute, Chengdu Academy of Agricultural and Forestry Sciences, Chengdu 611130, Sichuan, China Forestry Research Institute, Chengdu Academy of Agricultural and Forestry Sciences Chengdu China

**Keywords:** *
Ascomycetes
*, *
Colletotrichum
*, fungal diversity, morphology, new taxa, phylogeny, species complex

## Abstract

In recent years, the number of reported species of bamboo-inhabiting fungi worldwide has continued to increase, and the forest regions of Southwest China have become hotspots for the discovery and study of new fungal species. Sichuan Province has abundant, diverse, and widely distributed staple bamboo resources for giant pandas. The stable and healthy bamboo forest ecosystems provide favorable habitats for the colonization and proliferation of bamboo-inhabiting fungi. In the present study, dead bamboo culms were collected from the Giant Panda National Park in Ya’an City and Aba Prefecture, Sichuan Province, China. Based on morphological characteristics and multilocus phylogenetic analyses (ITS, *act*, *chs-1*, *his3*, *gapdh*, and *tub2*), two new species, *Colletotrichum abaense* and *C. phyllostachydis*, are described in this paper. *Colletotrichum* is a globally widespread fungal genus. Most species in this genus are important plant pathogens, while some can colonize hosts as endophytes or saprobes. This genus comprises multiple species complexes and harbors abundant cryptic species. The discovery of the two new species adds to the germplasm resources and species diversity of bamboo-associated *Colletotrichum* in China. In this study, detailed morphological descriptions, illustrations, and comparisons with closely related species are provided. The findings lay a foundation for the exploration of bamboo fungal resources and the taxonomic study of the genus *Colletotrichum*.

## Introduction

Bamboos belong to the subfamily *Bambusoideae* of *Poaceae*. As multifunctional plants with outstanding ecological and economic values, they constitute a vital component of forest ecosystems. Serving as staple food resources, bamboos sustain the survival and reproduction of rare wild animals such as giant pandas, thus bearing unique conservation significance (Zhang et al. 2024). Sichuan Province hosts the core wild giant panda habitats and concentrated distributions of their staple bamboos, where various exclusive food bamboo species for giant pandas, including *Fargesia spathacea*, *Indocalamus latifolius*, *Qiongzhuea opienensis*, *Phyllostachys heteroclada*, and *Phyllostachys praecox*, are widely distributed. The health of bamboo communities directly determines the habitat stability and survival status of wild giant panda populations.

Fungi of the genus *Colletotrichum* represent a group of important phytopathogens characterized by broad host ranges and strong pathogenicity (Wang et al. 2025c). To date, reports on *Colletotrichum* infecting bamboos remain limited, with only a small number of records documenting bamboo-associated pathogens (Yang et al. 2024a). Given their robust pathogenic potential, these fungi pose latent threats to the stability of vegetation communities and the ecological health of giant panda habitats, highlighting an urgent need for research on early prevention and risk warning.

*Colletotrichum* is the sole genus of *Glomerellaceae*, *Glomerellales*, *Sordariomycetes*, and *Ascomycota* and is listed among the world’s top 10 most important phytopathogenic fungal genera (Dean et al. 2012). Members of this genus exhibit extensive host adaptability and cause severe crop losses. They infect multiple plant organs, including leaves, fruits, and stems, triggering anthracnose diseases that frequently lead to sharp yield reductions or total harvest failure across diverse crops (Cannon et al. 2012; Liu et al. 2020; Stutts and Vermerris 2020). The host–pathogen interactions of *Colletotrichum* are intricate: a single host plant may suffer co-infection by multiple *Colletotrichum* species, while one *Colletotrichum* species can parasitize a wide array of distinct hosts (Damm et al. 2012; Jayawardena et al. 2021).

Moreover, morphological traits of *Colletotrichum* fluctuate drastically with environmental conditions and host substrates, resulting in low stability of diagnostic morphological markers and complex host-associated phenotypic variations, which substantially impede accurate species identification (Hyde et al. 2009; Jayawardena et al. 2021). With continuous refinement of fungal taxonomic frameworks, numerous cryptic species of *Colletotrichum* have been discovered and assigned to distinct species complexes, among which well-characterized groups include the *C. orchidearum* species complex and the *C. boninense* species complex (Damm et al. 2019; Liu et al. 2022). The taxonomic classification of bamboo-associated *Colletotrichum* species has remained contentious for a long time. Based on a six-locus dataset (ITS, *act*, *chs-1*, *his3*, *gapdh*, and *tub2*), Liu et al. (2022) performed phylogenetic analyses using maximum likelihood (ML) and Bayesian inference (BA) and resolved the genus *Colletotrichum* into 16 species complexes and 15 singleton species. In their phylogenetic framework, *C. bambusicola*, *C. guangxiense*, *C. hsienjenchang*, *C. metake*, and *C. parabambusicola* clustered together as a distinct lineage, which supported the recognition of the *C. bambusicola* species complex.

Precise species delimitation and identification lay the fundamental foundation for exploring fungal diversity, unraveling host–pathogen interaction mechanisms and evolutionary trajectories, implementing precise plant disease management, and formulating phytosanitary quarantine standards. Traditional taxonomic frameworks for *Colletotrichum* were established primarily based on host association and morphological characters rather than natural evolutionary relationships. Such systems fail to reflect phylogenetic relatedness among taxa and severely restrict in-depth investigation and systematic research on the species diversity of the genus (Liu et al. 2022).

Currently, an integrative taxonomic approach combining multilocus molecular phylogeny, morphological observations, geographic distribution, and ecological traits has become the mainstream methodology for fungal taxonomy, enabling the construction of stable classification systems consistent with natural evolutionary patterns (Cai et al. 2009; Cannon et al. 2012; Marin-Felix et al. 2017; Jayawardena et al. 2020). The backbone phylogenetic framework of *Colletotrichum* has been largely resolved via multilocus phylogenetic analyses and continuously updated with the publication of new species (Bhunjun et al. 2021). The constant discovery of novel taxa further demonstrates that *Colletotrichum* harbors abundant undiscovered cryptic species, and the intricate structure of its species complexes creates major challenges for distinguishing new and cryptic taxa.

Accordingly, accurate identification of *Colletotrichum* species is an essential prerequisite for scientific early warning and efficient management of anthracnose diseases. Specialized research targeting *Colletotrichum* on staple bamboos of giant pandas is still scarce, with unclear baseline data on fungal species resources and incomplete taxonomic frameworks. Fungal resource surveys were carried out in the Giant Panda National Park in Ya’an City and Aba Prefecture, Sichuan Province, during which a number of bamboo-derived fungal specimens were collected. Their morphological characteristics are in close agreement with the diagnostic traits of the genus *Colletotrichum*. Based on morphological observations, multilocus phylogenetic analyses, and nucleotide sequence divergence comparisons, two novel species belonging to the *C. bambusicola* species complex are described and formally proposed herein. The findings enrich baseline data on fungi associated with giant panda staple bamboos and provide crucial references for species identification, diversity research, and habitat disease prevention of bamboo-inhabiting *Colletotrichum*.

## Materials and methods

### Sample collection and isolation

The specimens of the novel species involved in this study were collected from dead bamboo culms in April 2024 at the Giant Panda National Park in Ya’an City, Sichuan Province, and in July 2024 at the Wolong Giant Panda National Park in the Aba Tibetan and Qiang Autonomous Prefecture, Sichuan Province. After preliminary processing, field-collected specimens were packaged in paper envelopes or resealable plastic bags, labeled with basic information such as collection number and date, and subsequently transported to the laboratory, where they were stored in a refrigerator at 4 °C until use (Senanayake et al. 2020). During sample processing, bamboo culms bearing distinct fungal structures were examined under a stereomicroscope, and a direct-picking method was employed. Briefly, ascomata or pycnidia were first cross-sectioned using a sterilized scalpel blade, after which the internal contents were precisely removed with a sterile needle and transferred into sterile water. The contents were thoroughly dispersed by agitation using the sterile needle, and then a sterile dropper was used to draw up the fungal-containing suspension and dispense it onto potato dextrose agar (PDA) plates (potato 200 g, dextrose 20 g, agar 20 g, and 1,000 mL sterile water), evenly spreading the suspension over the PDA surface (Cai et al. 2009). The plates were incubated at 25 °C or at room temperature for 12–24 h. After spore germination, individual germinated ascospores or conidia were sequentially transferred to fresh PDA plates using aseptic techniques. During the incubation period, the plates were examined daily to promptly eliminate contaminated samples. After a certain period of growth, to promote sporulation, mycelium was transferred to sterile bamboo leaf-bearing PDA plates and incubated under alternating conditions of 12 h of near-ultraviolet light and 12 h of darkness at 25 °C. Once the target colonies produced spores, the following procedures were carried out: first, an appropriate amount of mycelium was scraped into 1.5 mL sterile centrifuge tubes and stored at –20 °C for subsequent DNA extraction and sequencing analysis; simultaneously, the remaining colonies were transferred to cryovials containing PDA medium and maintained until the colonies completely covered the slant surfaces, after which they were preserved long-term by overlaying with liquid paraffin and sealing completely to ensure reliable strain preservation. The plant specimens collected in this study are deposited in the Mycological Herbarium of Sichuan Agricultural University (SICAU, Chengdu, China). The isolated strains are preserved in the Culture Collection Center of Sichuan Agricultural University (SICAUCC). Taxonomic information for the strains has been registered in accordance with international standards.

### Morphological observation

Under laboratory conditions, specimens were first subjected to preliminary screening and macroscopic morphological observation using a Nikon SMZ745 stereomicroscope, followed by micromorphological examination of fungal structures using an Olympus BX43 biological microscope. Representative specimens were selected based on fungal morphological characteristics and colony growth on natural substrates. The selected representative specimens were then subjected to high-resolution microscopic observation and image capture using a BX53 differential interference contrast microscope. For ascomycetous taxa, particular attention was given to observing and recording ascomatal morphology (including shape, size, and color), peridial structure, ascus arrangement, paraphysis morphology, and ascospore shape, size, surface ornamentation, and other features; for coelomycetes, emphasis was placed on observing conidiophore branching patterns, conidiogenous cell morphological characteristics, and conidial shape, size, septation, and other micromorphological features. All dimensional measurements of microstructures were performed using IFW software, with a minimum of 20 replicate measurements per feature to ensure data accuracy and reliability. Microscopic images were captured using a DS-Ri2 high-definition camera, and image processing and feature analysis were conducted using NIS-Elements imaging software.

### DNA extraction and PCR amplification

The 1.5 mL centrifuge tubes preserved in a –20 °C refrigerator were removed, after which liquid nitrogen was added to rapidly freeze the samples, and the mycelium was thoroughly ground into powder using a frozen grinding apparatus. Genomic DNA was extracted from the ground samples following the operating instructions of the plant genomic DNA extraction kit. Polymerase chain reaction (PCR) was employed to amplify six genetic loci, namely ITS, *chs-1*, *tub2*, *act*, *his3*, and *gapdh*. The primers and amplicon fragment sizes for each gene are listed in Table 1, and the reaction conditions are presented in Table 2. The PCR reaction volume was 25 μL (comprising 2 μL of genomic DNA, 1 μL each of forward and reverse primers, 8.5 μL of double-distilled water, and 12.5 μL of 2× Rapid Taq Master Mix). After amplification, the products were electrophoresed on 1% agarose gels to visualize band patterns. The PCR products were then sent to Tsingke Biotechnology Co., Ltd. for sequencing, and the returned sequencing results were assembled by splicing forward and reverse high-quality reads using BioEdit v. 7.0.5.3 software (Hall 2004). The newly generated sequence data in this study have been deposited in GenBank (https://www.ncbi.nlm.nih.gov). Sequences of the *Colletotrichum bambusicola* species complex used for constructing the multigene dataset were downloaded from GenBank (Table 2).

**Table 1.** PCR primers and amplified gene fragments.

**Gene**	**Primer**	**Primer sequence (5'-3')**	**Reference**
ITS	ITS1	TCCGTAGGTGAACCTGCGG	White et al. 1990
	ITS4	TCCTCCGCTTATTGATATGC	
*act*	ACT-512F	ATGTGCAAGGCCGGTTTCGC	Carbone and Kohn 1999
	ACT-783R	TACGAGTCCTTCTGGCCCAT	
*chs*-1	CHS-79F	TGGGGCAAGGATGCTTGGAAGAAG	Carbone and Kohn 1999
	CHS-345R	TGGAAGAACCATCTGTGAGAGTTG	
*gapdh*	GDF1	GCCGTCAACGACCCCTTCATTGA	Guerber et al. 2003
	GDR1	GGGTGGAGTCGTACTTGAGCATGT	
*his*3	CYLH3F	AGGTCCACTGGTGGCAAG	Crous et al. 2004
	CYLH3R	AGCTGGATGTCCTTGGACTG	
*tub*2	T1	AACATGCGTGAGATTGTAAGT	O’Donnell et al. 1998
	Bt2b	ACCCTCAGTGTAGTGACCCTTGGC	Glass and Donaldson 1995

**Table 2.** PCR amplification protocols for the six gene regions.

**Gene**	**Pre-denaturation**	**Thermal cycling (denaturation, annealing, extension)**	**Final extension**
ITS	94 °C: 3 min	(94 °C: 30 s, 55 °C: 50 s, 72 °C: 1 min) × 35 cycles	72 °C: 10 min
*chs*-1	94 °C: 3 min	(94 °C: 30 s, 55 °C: 50 s, 72 °C: 1 min) × 35 cycles	72 °C: 10 min
*act*	94 °C: 3 min	(94 °C: 30 s, 51 °C: 50 s, 72 °C: 1 min) × 35 cycles	72 °C: 10 min
*his*3	95 °C: 3 min	(95 °C: 30 s, 56 °C: 30 s, 72 °C: 45 s) × 35 cycles	72 °C: 10 min
*gapdh*	95 °C: 4 min	(95 °C: 30 s, 60 °C: 30 s, 72 °C: 45 s) × 35 cycles	72 °C: 7 min
*tub*2	94 °C: 3 min	(94 °C: 30 s, 51 °C: 50 s, 72 °C: 1 min) × 35 cycles	72 °C: 10 min

### Phylogenetic analysis

The assembled sequences were subjected to BLAST searches against the NCBI database, which revealed that the ITS gene sequences of the strains in this study were related to species of the genus *Colletotrichum*. Upon reviewing the literature, it was found that phylogenetic analyses of different species complexes within *Colletotrichum* employ different combinations of sequence fragments depending on the complex. Based on the ITS sequence results, the two newly collected species were similar to the *C. bambusicola* species complex; therefore, a combined sequence dataset comprising ITS, *act*, *chs-1*, *his3*, *gapdh*, and *tub2* was selected for analysis. In this study, sequence information for the relevant multigene fragments was compiled from GenBank (Table 3), and *C. nigrum* CBS 169.49 and *C. nigrum* CBS 128507 were selected as outgroup taxa. Phylogenetic analyses were performed on the downloaded sequence fragments using ML and Bayesian inference (BI) algorithms. First, all sequences were aligned using MAFFT version 7, with manual adjustments made when necessary (https://mafft.cbrc.jp/alignment/server/index.html, Katoh et al. 2019). The gene datasets were concatenated using Mesquite v. 3.11 software (http://www.mesquiteproject.org/) to construct a combined multigene matrix for subsequent phylogenetic analyses (Maddison and Maddison 2019). ML analyses were performed on the CIPRES dataset using RAxML-HPC2 (XSEDE 8.2.10) under the GTRGAMMA model, with bootstrap support (MLBP) assessed from 1,000 pseudoreplicates (Miller et al. 2010). BI was implemented in MrBayes v. 3.2.2 (Ronquist et al. 2012), and Bayesian posterior probabilities (BPP) were estimated via Markov chain Monte Carlo (MCMC) sampling as described by Tang et al. (2007). The optimal nucleotide substitution models for each locus were selected using MrModeltest v. 2.2 (Nylander 2004): SYM+I+G for ITS, SYM+G for *chs-1*, HKY+I for *act* and *tub2*, and HKY+G for *gapdh* and *his3*. The MCMC algorithm was run for 10 million generations, and after the run was completed, the resulting BI tree file (.con.tre) was downloaded. The BI tree was visualized and edited using FigTree 1.4.4 software. The ML tree topology was selected as the basis, and the resulting ML tree was visualized and edited using FigTree 1.4.4 software. The tree was then refined using Adobe Illustrator 2023 software (Yang et al. 2024a).

**Table 3.** GenBank accession numbers used in the phylogenetic analyses.

**Strain**	**Culture**	**GenBank accession number**					
		**ITS**	***gapdh***	***chs*-1**	***his*3**	***act***	***tub*2**
***Colletotrichum abaense***	**SICAUCC 26-0233T**	**PZ501536**	**PZ700614**	**PZ700609**	**PZ700618**	**PZ700605**	**PZ733948**
***C. abaense***	**SICAUCC 26-0234**	**PZ501537**	**PZ700615**	**PZ700610**	**PZ700619**	**PZ700606**	**PZ733949**
*C. bambusicola*	CNUCC 307307T	MT199632	MT192844	MT192871	N/A	MT188638	MT192817
*C. bambusicola*	CNUCC 310192	MT199624	MT192840	MT192867	N/A	MT188634	MT192813
*C. bambusicola*	CNUCC 305200	MT199619	MT192846	MT192873	N/A	MT188640	MT192819
*C. bambusicola*	CNUCC 30893	MT199621	MT192843	MT192870	N/A	MT188637	MT192816
*C. bambusicola*	LC 8469	MZ595857	MZ772869	MZ799334	MZ673877	MZ664155	MZ673978
*C. bambusicola*	LC 8498	MZ595859	MZ664097	MZ799335	MZ673879	MZ664157	MZ673980
*C. bambusicola*	LC 8468	MZ595856	MZ664095	MZ799333	MZ673876	MZ664154	MZ673977
*C. bambusicola*	LC 8533	MZ595862	MZ664096	MZ799336	MZ673882	MZ664160	N/A
*C. parabambusicola*	LC 13883	MZ595902	N/A	MZ799337	MZ673922	MZ664200	MZ674020
*C. parabambusicola*	LC 13884T	MZ595904	MZ664098	MZ799338	MZ673924	MZ664202	MZ674022
*C. parabambusicola*	CNUCC 3041	MT199616	MT192849	MT192876	N/A	MT188643	MT192822
*C. parabambusicola*	CNUCC 302410	MT199609	MT192856	MT188623	N/A	MT188650	MT192829
*C. parabambusicola*	CNUCC 303223	MT199613	MT192852	MT188619	N/A	MT188646	MT192825
*C. parabambusicola*	CNUCC 303162	MT199614	MT192851	MT192878	N/A	MT188645	MT192824
*C. metake*	MAFF 244029	OK090429	N/A	N/A	N/A	N/A	OK236390
*C. metake*	MAFF 243970	OK090430	N/A	N/A	N/A	N/A	OK236391
*C. metake*	MAFF 241800	OK090431	N/A	OK236386	N/A	OK236388	OK236392
*C. guangxiense*	CNUCC 310138T	MT199633	MT192834	MT192861	N/A	MT188628	MT192805
*C. guangxiense*	CNUCC 307305	MT199625	MT192839	MT192866	N/A	MT188633	MT192807
*C. guangxiense*	CNUCC 305268	MT199626	MT192838	MT192865	N/A	MT188632	MT192808
*C. guangxiense*	CNUCC 310185	MT199627	MT192837	MT192864	N/A	MT188631	MT192809
*C. guangxiense*	CNUCC 305258	MT199628	MT192836	MT192863	N/A	MT188630	MT192810
*C. guangxiense*	CNUCC 310181	MT199629	MT192835	MT192862	N/A	MT188629	MT192806
*C. guangxiense*	CNUCC 305238	MT199630	MT192833	MT192860	N/A	MT188627	MT192811
*C. guangxiense*	CNUCC 307304	MT199631	MT192832	MT192859	N/A	MT188626	MT192812
*C. hsienjenchang*	MAFF 243051	AB738855	N/A	AB738846	AB738847	AB738845	N/A
***C. phyllostachydis***	**SICAUCC 26-0231T**	**PZ501533**	**PZ700611**	**PZ700607**	**PZ700616**	**PZ700603**	**PZ733946**
***C. phyllostachydis***	**SICAUCC 26-0232**	**PZ501534**	**PZ700612**	**PZ700608**	**PZ700617**	**PZ700604**	**PZ733947**
*C. nigrum*	CBS 169.49	JX546838	JX546742	JX546693	JX546791	JX546646	JX546885
*C. nigrum*	CBS 128507	JX546843	JX546747	JX546698	JX546796	JX546651	JX546890

Note: Newly generated sequences are in bold. “T” indicates ex-type strains. “N/A” indicates unavailable data in GenBank.

## Results and analysis

### Phylogenetic analysis

The phylogram generated from ML analysis based on the combined ITS, *act*, *chs-1*, *his3*, *gapdh*, and *tub2* sequence dataset represents *Colletotrichum*. The combined analyses included 32 strains and comprised 2,716 characters, including gaps (616 characters for ITS, 300 characters for *act*, 300 characters for *chs-1*, 425 characters for *his3*, 301 characters for *gapdh*, and 774 characters for *tub2*). *C. nigrum* CBS 169.49 and *C. nigrum* CBS 128507 were selected as the outgroup taxa. Phylogenetic trees generated from ML and BI analyses were similar in overall topology. The best-scoring RAxML tree, with a final likelihood value of –8,757.690199, is presented. The matrix had 771 distinct alignment patterns, with 27.73% undetermined characters or gaps. Estimated base frequencies were as follows: A = 0.217783, C = 0.313600, G = 0.246607, *T* = 0.222010; substitution rates AC = 1.064066, AG = 3.027190, AT = 1.266099, CG = 0.986256, CT = 3.952330, GT = 1.000000; tree length = 0.525511; gamma distribution shape parameter α = 0.255921. Bayesian posterior probabilities (BPP) from MCMC were evaluated with a final average standard deviation of split frequencies of less than 0.01.

### Genealogical concordance phylogenetic species recognition analysis

Genealogical concordance phylogenetic species recognition is a useful approach for species delimitation, as it can efficiently and accurately detect significant recombination events within datasets, as emphasized by Quaedvlieg et al. (2014). In this study, the capabilities of SplitsTree V4 software were used to conduct an in-depth analysis of the data through the pairwise homoplasy index (PHI) test. This test evaluates recombination among closely related taxa. A PHI value below the threshold of 0.05 (*Φw* < 0.05) clearly indicates the presence of significant recombination events in the dataset. To ensure the accuracy and comprehensiveness of the analysis, a concatenated multigene dataset encompassing all closely related species was selected. This dataset provides a more reliable genetic foundation for the study. A split graph was constructed using LogDet transformation and split decomposition options, effectively visualizing the complex genetic relationships among the species under investigation.

### Taxonomy

#### Colletotrichum abaense

Taxon classificationFungiGlomerellalesGlomerellaceae

F.H. Wang & C.L. Yang sp. nov.

EFABE938-9C57-5255-8DFF-32ED24422B5B

Fungal Names: FN 905606

Fig. 1

##### Etymology.

The species epithet “abaense” is named after the geographic location of the collection site, Aba Prefecture, Sichuan Province, China.

**Figure 1.**
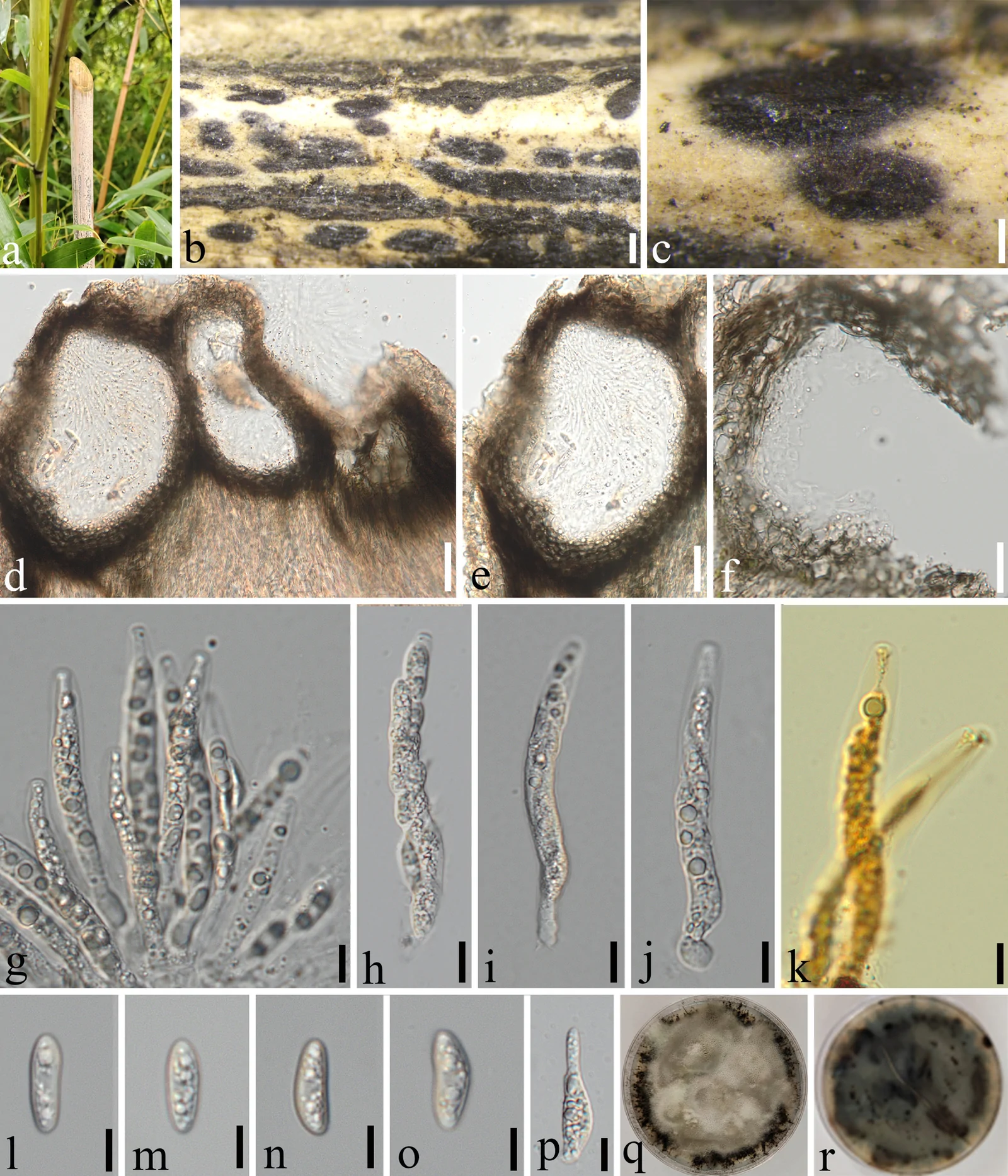
*Colletotrichum abaense* (SICAU 26-0324, holotype). **a–c**. Stromata on host surface; **d**. Longitudinal section of stromata; **e**. Longitudinal section of ascoma; **f**. Peridium; **g**. Paraphyses and asci; **h–j**. Asci; **k**. Asci stained with Melzer’s reagent; **l–o**. Ascospores; **p**. Germinated ascospore; **q, r**. Obverse and reverse views of colony on PDA. Scale bars: 500 μm (**b**); 200 μm (**c**); 60 μm (**d**); 50 μm (**e**); 20 μm (**f–k**); 10 μm (**l–p**).

##### Holotype.

SICAU 26-0324.

##### Habitat.

On the culm of *Phyllostachys heteroclada*.

##### Description.

***Sexual morph*: *Ascomata*** 240–300 × 150–240 μm (x– = 240 × 195, *n* = 20), perithecial, substomatal, solitary or gregarious, superficial or immersed, multilocular, subglobose to globose or irregular in shape, with ostiole and ostiolar setae. ***Peridium*** 19–34 μm thick, composed of several layers of brownish to dark brown flattened angular cells, with inner cells hyaline to pale brown. ***Paraphyses*** hyaline, smooth, 90–110 × 1.5–3 μm (x– = 100 × 2, *n* = 20), unbranched. ***Asci*** 50–88 × 6–14 μm (x– = 70 × 10 μm, *n* = 20), unitunicate, 8-spored, clavate to cylindrical or broadly clavate, with a short stipe, apically rounded, with an apical chamber, amyloid (Meltzer’s reaction positive). ***Ascospores*** 10–14 × 6–8 μm (x–= 12 × 7, *n* = 20), uniseriate, aseptate, hyaline, smooth-walled, with small guttules, fusiform or reniform, with both ends rounded. Appressoria not observed. ***Asexual morph***: Undetermined.

##### Culture characteristics.

Ascospores germinated normally in sterile water, producing germ tubes from both ends after 12 h of incubation at 25 ± 1 °C and 95% relative humidity. Growth was relatively rapid, with colony diameter reaching 60 mm on PDA after 8 d at 25 °C. Colonies were subcircular in shape with regular margins, with abundant aerial mycelium that was grayish-white, floccose, and growing radially at the edges; no pigmentation was produced. After 14 d, the mycelium turned grayish-brown with olivaceous margins, and the reverse side was dark brown.

##### Material examined.

Sichuan Province, Aba Prefecture, Wenchuan County, Wolong Town, saprobic on dead culms of *Phyllostachys heteroclada*, (31°0'44.22"N, 103°9'33.23"E, Alt 2,040 m) 9 July 2024, Wang Feihu, WFH202407015 (SICAU 26-0324, holotype), ex-type culture SICAUCC 26-0233. ibid. WFH202407015B (SICAU 26-0325, paratype), living culture SICAUCC 26-0234.

##### GenBank accession numbers.

SICAUCC 26-0233 (ITS: PZ501536; *act*: PZ700605; *chs-1*: PZ700609; *gapdh*: PZ700614; *his3*: PZ700618; *tub2*: PZ733948); SICAUCC 26-0234 (ITS: PZ501537; *act*: PZ700606; *chs-1*: PZ700610; *gapdh*: PZ700615; *his3*: PZ700619; *tub2*: PZ733949).

##### Notes.

**Figure 2.**
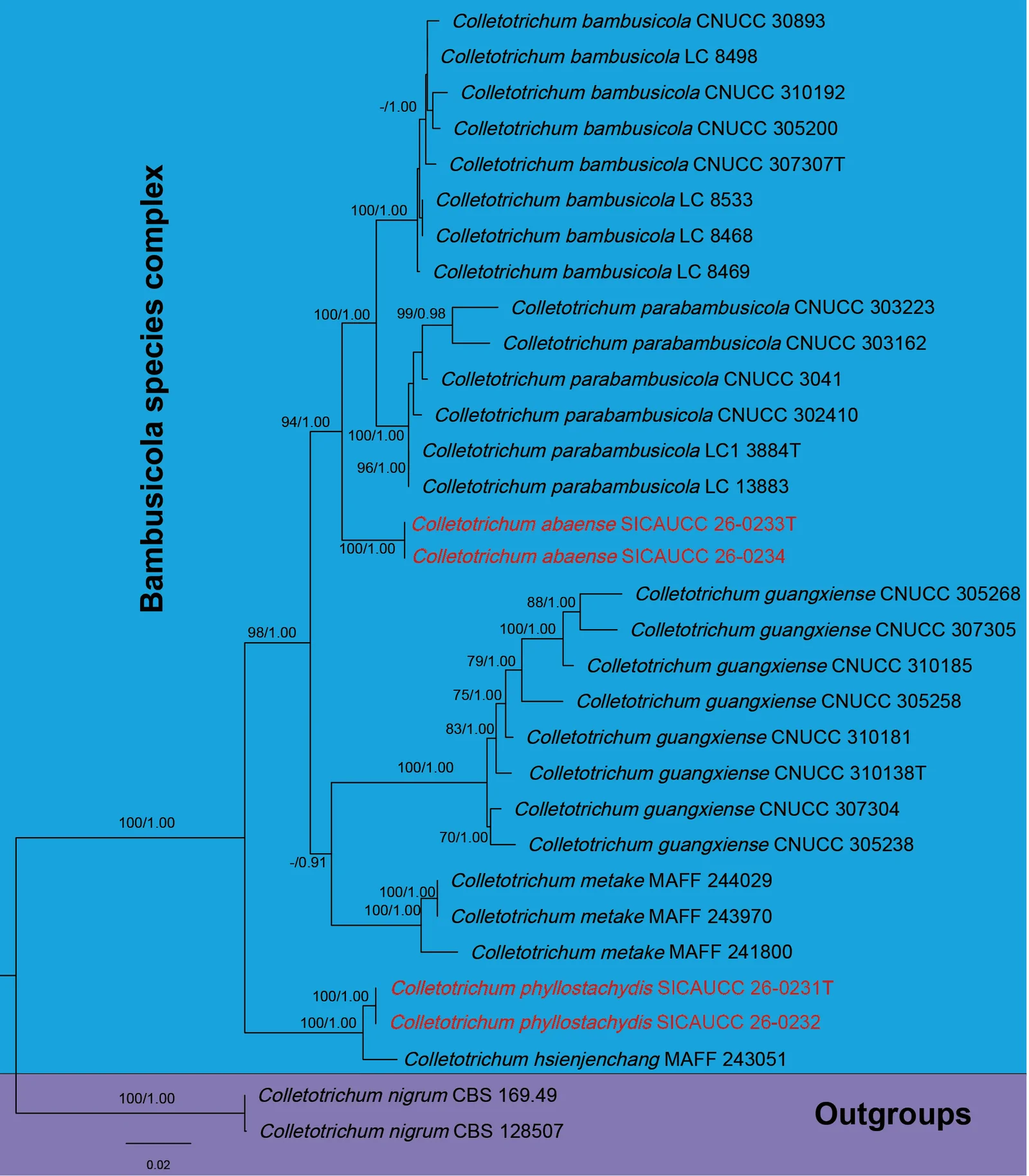
Bootstrap support values for ML greater than 70% and BYPP greater than 0.90 are shown above the nodes as MLBS/BPP. Type strains are indicated by the letter “T,” and newly generated sequences are shown in bold red.

**Figure 3.**
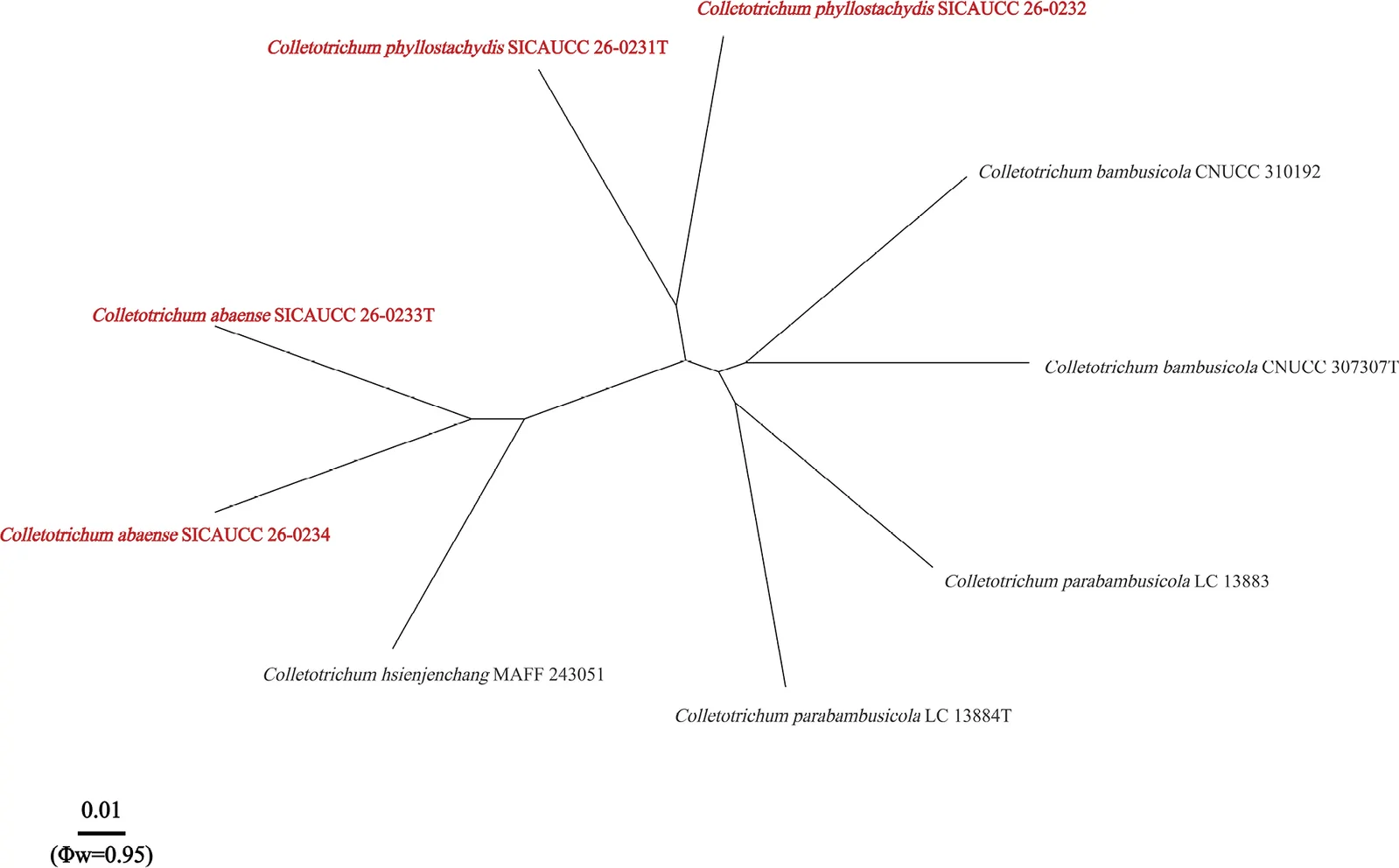
The splits graph from the pairwise homoplasy index (PHI) test generated from the concatenated gene set of ITS, *act*, *chs-1*, *his3*, *gapdh*, and *tub2* sequence data of closely related species of the *Colletotrichum bambusicola* species complex using both LogDet transformation and splits decomposition. The PHI test result (*Фw*) = 0.95) indicates the absence of recombination in the dataset. The strains obtained in this study are in red. The ex-type strains are marked with “T” after the strain number label.

Phylogenetic analyses revealed that the newly collected strains in this study (SICAUCC 26-0233, SICAUCC 26-0234) formed a distinct clade (Fig. 2), closely related to *Colletotrichum parabambusicola* and *C. bambusicola*. Morphologically, both *C. parabambusicola* and *C. bambusicola* possess appressoria, and their ascospores lack guttules, whereas the strains in this study lacked appressoria and contained guttules within the ascospores (Liu et al. 2022). Pairwise base comparisons showed that between the type strain of this study (SICAUCC 26-0233) and *C. parabambusicola*, the nucleotide differences for *gapdh*, *chs-1*, *his3*, and *act* gene sequences were 8.7% (25/286 bp, 0 gaps), 1.7% (5/288 bp, 0 gaps), 7% (27/388 bp, 7 gaps), and 2.2% (6/278 bp, 0 gaps), respectively. Between the type strain of this study (SICAUCC 26-0233) and *C. bambusicola*, the nucleotide differences for *gapdh*, *chs-1*, and *act* gene sequences were 11.9% (34/286 bp, 3 gaps), 2.1% (6/288 bp, 0 gaps), and 3.6% (10/278 bp, 1 gap), respectively. The PHI test results indicated no significant recombination events between this strain and its closely related taxa (*Φw* = 0.95) (Fig. 3). Both morphological characteristics and molecular phylogenetic analyses support the recognition of this fungus as a novel species.

#### Colletotrichum phyllostachydis

Taxon classificationFungiGlomerellalesGlomerellaceae

F.H. Wang & C.L. Yang sp. nov.

408EABE2-6660-5A61-9939-156EC7FD8475

Fungal Names: FN 905607

Fig. 4

##### Etymology.

The specific epithet “phyllostachydis” refers to the host genus *Phyllostachys* from which the fungus was collected.

**Figure 4.**
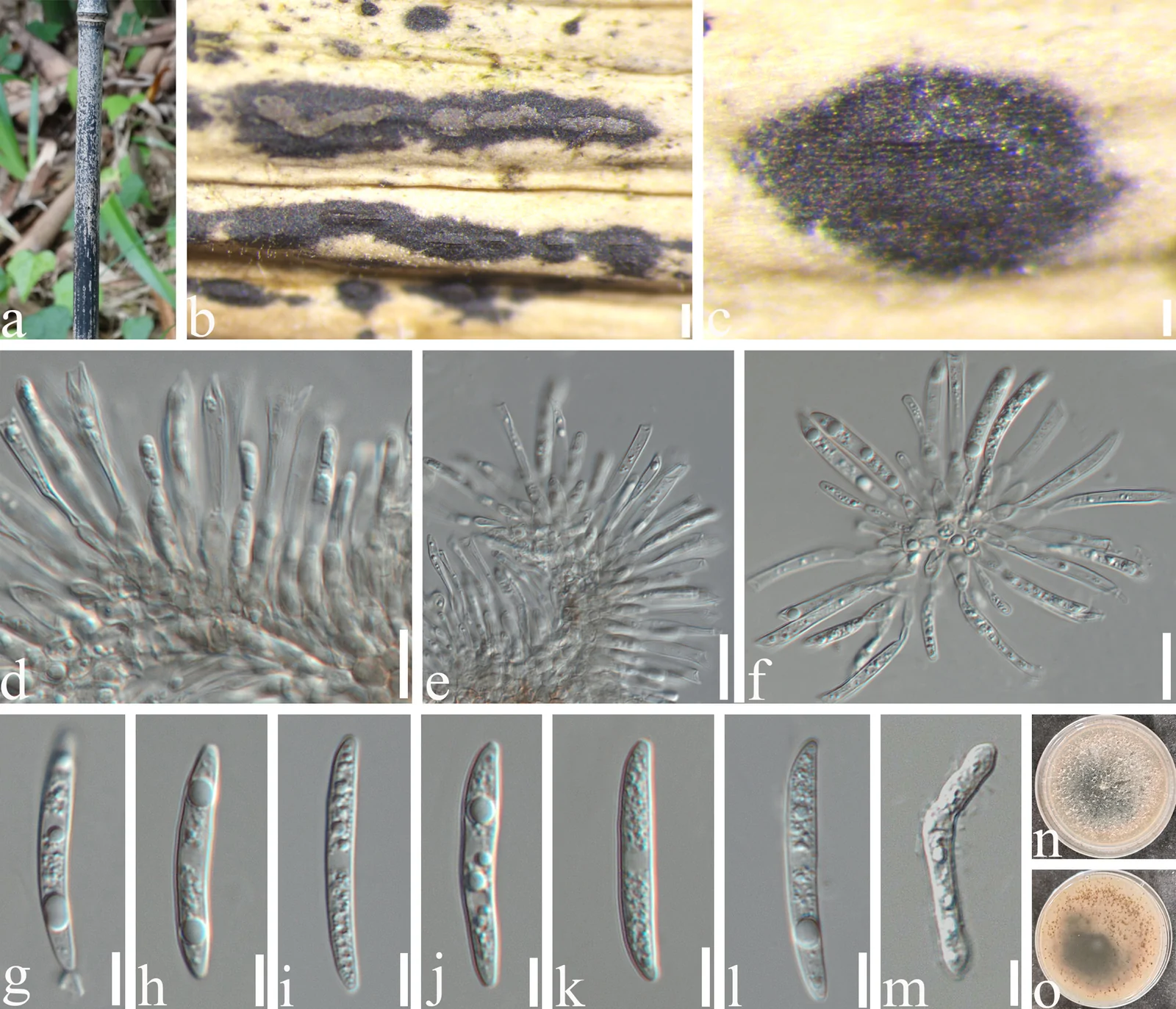
*Colletotrichum phyllostachydis* (SICAU 26-0322, holotype). **a–c**. Stromata on natural substrate; **d–f**. Conidiophore, conidiogenous cells giving rise to conidia; **g–l**. Conidia; **m**. Germinated conidium; **n, o**. Obverse and reverse views of colony on PDA. Scale bars: 500 μm (**b**); 80 μm (**c**); 20 μm (**d–f, m**); 10 μm (**g–l**).

##### Holotype.

SICAU 26-0322.

##### Habitat.

On the culm of *Phyllostachys heteroclada*.

##### Description.

***Sexual morph***: Undetermined. ***Asexual morph*: *Conidiomata*** 240–310 × 216–278 μm (x– = 270 × 240 μm, *n* = 20), pustulate, brown to dark brown, subglobose to ellipsoidal, immersed, solitary or gregarious. ***Conidiophores*** 11–17 × 3–7 μm (x– = 14 × 4 μm, *n* = 20), arising from a cushion-shaped structure composed of hyaline to pale brown cells, hyaline to pale brown, septate, occasionally branched. Setae not observed. ***Conidiogenous cells*** 16–24 × 4–8 μm (x– = 20 × 6 μm, *n* = 20), hyaline, smooth-walled, phialidic, cylindrical or flask-shaped, aseptate. ***Conidia*** 24–32 × 4–8 μm (x– = 28 × 6 μm, *n* = 30), hyaline, aseptate, smooth-walled, falcate, cylindrical, ellipsoidal or irregular in shape, with obtuse apices, containing guttules. Appressoria not observed.

##### Culture characteristics.

Conidia germinated normally in sterile water, producing germ tubes from both ends after 12 h of incubation at 25 ± 1 °C and 95% relative humidity. Growth was relatively rapid, with colony diameter reaching 60 mm on PDA after 12 d at 25 °C. Colonies were subcircular in shape with regular margins, with sparse aerial mycelium that was white and growing radially at the edges. After 18 d, the mycelium turned reddish-brown, and the reverse side was brown to reddish-brown.

##### Material examined.

Sichuan Province, Ya’an City, Yucheng District, saprobic on dead culms of *Phyllostachys heteroclada*, (29°52'28.28"N, 103°2'36.53"E, Alt 776 m), 27 April 2024, Wang Feihu, WFH202404006 (SICAU 26-0322, holotype), ex-type culture SICAUCC 26-0231. ibid. WFH202404006B (SICAU 26-0323, paratype), living culture SICAUCC 26-0232.

##### GenBank accession numbers.

SICAUCC 26-0231 (ITS: PZ501533; *act*: PZ700603; *chs-1*: PZ700607; *gapdh*: PZ700611; *his3*: PZ700616; *tub2*: PZ733946); SICAUCC 26-0232 (ITS: PZ501534; *act*: PZ700604; *chs-1*: PZ700608; *gapdh*: PZ700612; *his3*: PZ700617; *tub2*: PZ733947).

##### Notes.

Phylogenetic analyses revealed that the newly collected strains in this study (SICAUCC 26-0231, SICAUCC 26-0232) formed a clade with *C. hsienjenchang* MAFF 243051 (Fig. 2). Morphologically, according to previous records, the strain of *C. hsienjenchang* possesses distinct setae and appressoria, whereas the strains in this study lacked both setae and appressoria. The new species produces falcate or cylindrical conidia, and its conidial characteristics are similar to those of *C. hsienjenchang* (Sato et al. 2012). Pairwise base comparisons showed that between the type strain of this study (SICAUCC 26-0231) and *C. hsienjenchang* MAFF 243051, the nucleotide differences for *chs-1* and *his3* gene sequences were 2.5% (5/198 bp, 3 gaps) and 3.2% (12/371 bp, 1 gap), respectively. The PHI test results indicated no significant recombination events between this strain and its closely related taxa (*Φw* = 0.95) (Fig. 3). Both morphological characteristics and molecular phylogenetic analyses support the recognition of this fungus as a novel species.

## Discussion

Bamboo possesses substantial economic and edible value, rendering it a valuable natural resource. The utilization of bamboo by humans has a long history, and its extensive range of applications, diversity of developed products, and high economic returns are scarcely matched by conventional tree species. Moreover, bamboo species serving as the staple food of giant pandas hold even greater ecological significance, as they constitute the primary dietary resource for this species and are therefore critical to its conservation (Wang et al. 2025a; Wang et al. 2025b). To date, major bamboo diseases include bamboo shoot blight, rhombic leaf spot of *Phyllostachys heteroclada*, sooty mold, and tar spot disease, whereas diseases of bamboo caused by *Colletotrichum* fungi have been relatively infrequently documented (Yang et al. 2024a).

The genus *Colletotrichum* is recognized as one of the most significant groups of phytopathogenic fungi, capable of inciting anthracnose and other destructive diseases across a broad spectrum of economically important hosts worldwide (Dean et al. 2012). Over the past decade, research on the global species diversity of *Colletotrichum* has advanced progressively, with novel species being continually discovered and described, thereby contributing to a steady increase in the number of known species within the genus. To date, more than 300 *Colletotrichum* species have been reported globally; however, complete multigene sequence data remain unavailable for certain species, and phylogenetic support for several closely related species complexes remains suboptimal (Weir et al. 2012; Norphanphoun and Hyde 2023; Gomdola et al. 2025). This limitation is primarily attributable to incomplete gene fragment datasets and the paucity of molecular marker information for most closely related taxa. With the ongoing advancement of molecular technologies, molecular data have become indispensable for resolving taxonomic ambiguities within *Colletotrichum* (Marin-Felix et al. 2017; Bhunjun et al. 2021; Jayawardena et al. 2021; Liu et al. 2022; Aumentado et al. 2024). In this study, multilocus phylogenetic analyses using ITS, *gapdh*, *act*, *chs-1*, *tub2*, and *his3* molecular markers significantly improved species resolution. To evaluate whether recombination might obscure the phylogenetic relationships and species boundaries within the bamboo-associated *Colletotrichum* species complex, the pairwise homoplasy index (PHI) test was performed on the concatenated six-locus dataset. When *C. abaense* and *C. phyllostachydis* were compared with their respective closely related taxa, the PHI test yielded *Φw* values of 0.95 for both, substantially exceeding the 0.05 significance threshold. These results indicate no statistically significant recombination events between the two novel species and their phylogenetic neighbors, confirming that the concatenated dataset is free from reticulate evolutionary signals that could confound phylogenetic inference (Quaedvlieg et al. 2014). Their taxonomic status was further validated by the genealogical concordance phylogenetic species recognition (GCPSR) approach, providing strong evidence for delimiting these two species as distinct taxa within the *C. bambusicola* species complex. Within this genus, the *C. bambusicola* species complex comprises numerous morphologically similar yet genetically distinct taxa, which are commonly associated with bamboo hosts.

China, characterized by its extensive territory and abundant host plant resources, harbors considerable *Colletotrichum* fungal diversity, as evidenced by the substantial number of species already reported from the country. *Colletotrichum* fungi exhibit a broad host range, infecting various herbaceous and woody plants, and can also be isolated from diverse substrates, including soil and aquatic environments. Among them, the majority of species predominantly parasitize crops, medicinal plants, and ornamental plants, representing a typical group of plant-pathogenic fungi (Dentika et al. 2023; Talhinhas and Baroncelli 2023; Yang et al. 2024b; Wang et al. 2025c). In the present study, two novel *Colletotrichum* species were isolated from bamboo; however, their pathogenicity remains to be further elucidated through additional observations and experimental investigations.

Current research on *Colletotrichum* has predominantly focused on species identification and disease surveys, whereas studies addressing their secondary metabolites, bioactivities, and potential applications remain comparatively underexplored. Previous investigations have indicated that certain *Colletotrichum* species are capable of producing diverse bioactive metabolites with promising potential for biological control and pharmaceutical development. Consequently, continued efforts in the exploration of *Colletotrichum* resources, species delineation, and functional characterization are expected to uncover additional taxa with potential applications in agriculture and biopharmaceutical development, thereby providing a theoretical foundation for the scientific management of plant anthracnose and the sustainable utilization of fungal resources.

## Supplementary Material

XML Treatment for Colletotrichum abaense

XML Treatment for Colletotrichum phyllostachydis

## Additional information

### Artificial Intelligence (AI) use

The authors accept full responsibility for the content of the manuscript, including the disclosure of any use of AI.

No AI tools were used in the preparation of this manuscript.

### Author ORCIDs

F. Wang https://orcid.org/0000-0002-3227-5194

F. Liu https://orcid.org/0000-0003-4580-7169

X. Xu https://orcid.org/0000-0002-6832-5421

C. Yang https://orcid.org/0000-0002-3854-8835

### Data availability

All of the data that support the findings of this study are available in the main text.
